# Morphology and multiparameter flow cytometry combined for integrated lymphoma diagnosis on small volume samples: possibilities and limitations

**DOI:** 10.1007/s00428-024-03819-3

**Published:** 2024-05-28

**Authors:** Mats Ehinger, Marie C. Béné

**Affiliations:** 1 Division of Pathology, Department of Clinical Sciences Lund, Lund University, Lund, Sweden; 2 Faculty of Medicine, Nantes University, Nantes, France; 3 Department of Clinical Genetics, Pathology and Molecular Diagnostics, Office for Medical Services, Region Skåne, Lund, Sweden

**Keywords:** Cytology, Biopsy, Fine needle, Flow cytometry, Lymphoma, Diagnosis

## Abstract

The diagnosis of lymphoma relies mainly on clinical examination and laboratory explorations. Among the latter, morphological and immunohistochemical analysis of a tissue biopsy are the cornerstones for proper identification and classification of the disease. In lymphoma with blood and/or bone marrow involvement, multiparameter flow cytometry is useful. This technique can also be applied to fresh cells released from a biopsy sample. For full comprehension of lymphomas, surgical biopsies are best and indeed recommended by the hematopathological community. Currently, however, there is a global trend towards less invasive procedures, resulting in smaller samples such as core needle biopsies or fine needle aspirations which can make the diagnosis quite challenging. In this review, the possibilities and limitations to make an accurate lymphoma diagnosis on such small volume material are presented. After recalling the major steps of lymphoma diagnosis, the respective value of histology, cytology, and flow cytometry is discussed, including handling of small specimens. The benefits of an integrated approach are then evoked, followed by discussion about which attitude to adopt in different contexts. Perhaps contrary to the prevailing view among many pathologists, a full diagnosis on small volume material, combined with relevant ancillary techniques, is often possible and indeed supported by recent literature. A glimpse at future evolutions, notably the merit of artificial intelligence tools, is finally provided. All in all, this document aims at providing pathologists with an overview of diagnostic possibilities in lymphoma patients when confronted with small volume material such as core needle biopsies or fine needle aspirations.

## What are the steps of lymphoma diagnosis?

Suspicion of a lymphoproliferative disorder can occur in the presence of highly suggestive clinical signs of enlarged lymph nodes or spleen, sometimes self-discovered by the patient. Clinical features can be less specific such as asthenia and cytopenia. It can also be the chance observation of lymphocytosis on a routine complete blood count (CBC) or of an immunoglobulin peak at serum protein electrophoresis, another very commonly prescribed laboratory test. Physical examination will ensue, followed if needed by a CBC and morphological observation of the lymphocytes in the cases of peripheral blood involvement. Multiparameter flow cytometry (MFC) has its place here as a complement to morphology. Extended explorations through bone marrow aspiration will also include morphological and immunophenotypic examinations. Meanwhile, imaging will have been organized, allowing for a full appreciation of the tumoral mass and choice of the site for the collection of material to send to the pathologist for a full diagnosis by morphological observation and immunohistochemistry (IHC). Cytogenetics by fluorescence in situ hybridization (FISH) can also be performed at this point as well as molecular explorations. Of course, the latter two can also be applied earlier on peripheral or bone marrow cells.

The key question however is the type of sample that will be sent for hematopathology. Surgical excision (SE) of lymphadenopathy is indeed the gold standard according to guidelines from leading societies and cancer centers such as the European Society for Medical Oncology (ESMO) and the National Comprehensive Cancer Network (NCCN). Yet, recommendation is one thing and reality often another. In real life, a core needle biopsy (CNB) is often the first choice even for easily accessible lymph nodes from the neck, groin, or axillae [1]. At least in Western countries, the trend is somewhat deceptive with more CNBs being performed at the expense of SEs. For example, in a recent French survey of more than 30,000 biopsies interrogated for lymphoma, CNBs increased from 25% of the specimens in 2010 to 40% in 2018 [2]. A similar study from the Kiel registry in Germany [3] points in the same direction and corroborates the gut feeling among many hematopathologists that there is less and less material to deal with. The COVID-19 pandemic has probably aggravated the problem with clinicians naturally less prone to bring about excisional biopsies during the pandemic [4]. As oncologists and surgeons were still getting diagnoses during the pandemic, despite increasing reliance on small volume biopsies (SVBs), the number of SEs may not easily revert even to the modest pre-COVID-19 levels. The trend with less diagnostic material is confronted with ever-increasing demands from patients and clinicians. Reality is brutal, and even though hematopathologists strongly advocate excisional biopsies, it will be necessary to adopt and develop strategies to deal with SVBs. These include fine needle aspirations (FNAs) that in certain situations and in combination with ancillary techniques can be very useful for an actionable diagnosis. Yet, before digging into controversies regarding the diagnostic possibilities and limitations with SVBs, it is important to recognize that hematopathologists cannot do magic with limited material.

## What can hematopathologists do with small volume material?

**Fig. 1:**
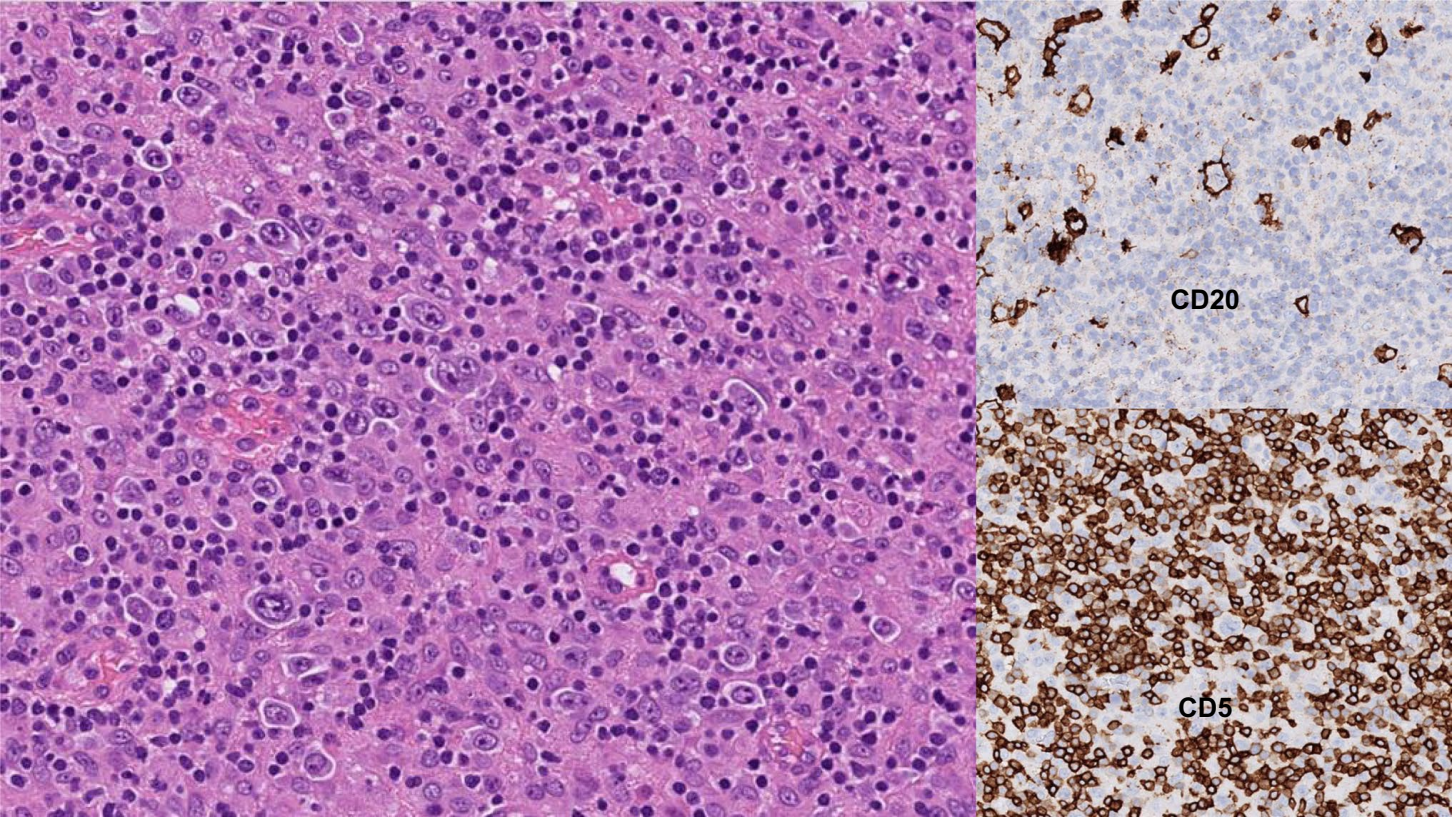
Immunoarchitecture is necessary to diagnose TCHRLBCL. Scattered large CD20+ B-cells can be seen against a background of numerous T-lymphocytes (CD5+) and histiocytes (hematoxylin-eosin)

Big is beautiful, and pathologists have an everlasting reputation for craving for more tissue, not the least in the field of lymphoma diagnosis. The main reason for the hunger of big chunk biopsies is that the tissue architecture provides crucial clues for many types of diagnosis. This is particularly true for Hodgkin lymphomas (HLs), T-cell histiocyte-rich large B-cell lymphoma (TCHRLBCL) (Fig. 1), and peripheral T-cell lymphomas, notably angioimmunoblastic T-cell lymphoma (AITL) [2]. Both HL, TCHRLBCL, and AITL tend to have a large inflammatory component which contributes to the diagnostic difficulties on SVBs. We will first discuss CNBs and in the next section turn our attention to FNAs.

How bad are CNBs as compared to SEs to make a reliable diagnosis? In fact, an accurate diagnosis on CNBs (combined with IHC) can be made in as many 92% of cases, as compared to 98% on SEs [2]. Uncertainty as to the diagnosis of lymphoma vs reactive lymphadenopathy or inability to render a specific lymphoma subtype diagnosis were the most common causes for lack of definitive diagnosis in this huge study comprising 10.285 CNBs.^2^ Notably, all diagnoses were made by expert hematopathologists, and it was wisely concluded that whenever pathologists feel uncertain about a diagnosis of lymphoma on a CNB, SE should be recommended. In three earlier studies examining CNBs guided with various imaging techniques including 45, 346, and 153 lymphoma samples, respectively, a specific lymphoma diagnosis was possible in 96%, 93%, and 99% of the cases [5–7]. In two quite large studies from North America with 263 and 191 CNBs, respectively, a specific subtype diagnosis was possible in 75% and 81% of the cases with lymphoma [8, 9]. SE as reference gold standard was available in 38 and 60 cases, respectively, where a diagnosis of lymphoma or of a benign condition had been made on the corresponding CNB. Among these paired samples, there were no false positives and only one false negative in each study, translating to high positive and negative predictive values (Table 1). The two missed diagnoses were a classic HL in one study and a diffuse large B-cell lymphoma (DLBCL) in the other [8, 9]. Although adding information from MFC from corresponding FNA generally helped in establishing a lymphoma diagnosis, it did not significantly improve subclassification [8]. Taken together, these data imply that it is possible to make a definitive lymphoma diagnosis on most CNBs.

**Table 1:** Predictive values and concordance rates of SVB with or without MFC for diagnosing and subtyping lymphomas

	Type of SVB	MFC	Lymphoma	Number of cases (lymphomas/not lymphomas)	Se/Sp	PPV	NPV	Concordance with reference histology (%)	Reference histology
Tomozawa et al. [5]	CNB	No	Lymphoma NOS	52 (45/7)	93/93	ND	100		SE and CFU
Cohen et al. [6]	CNB	No	Lymphoma NOS	541 (346/195)	92/ND	ND	98		Repeat CNB and CFU
Pugliese et al. [7]	CNB	No	Lymphoma NOS	164 (151/13)	99/ND	ND	85		SE and CFU
Amador-Ortiz et al. [8]	CNB+FNA	Yes	Lymphoma NOS	38 (29/9)	97/100	100	90		SE
Jelloul et al. [9]	CNB+FNA	Yes	Lymphoma NOS	60 (37/23)	ND	100	96		SE
Dong et al. [10]	FNA	Yes	NHL NOS	139	ND	100	ND		SE or CNB
			BL	2				100	
			MCL	1				100	
			SLL	9				100	
			LPL	3				100	
			MZL	6				33	
			DLBCL	21				95	
			FL NOS	13				100	
			PTCL	3				100	
Colorado et al. [11]	FNA	Yes	Lymphoma NOS	223 (130/93)	87/96^$^	98	93*		SE
			BL	4				75	
			MCL	8				75	
			SLL	7				100	
			LPL	1				100	
			MZL	1				100	
			DLBCL	22				82	
			FL NOS	35				91	
			ALCL	4				75	
			PL	1				100	
Schmid et al. [12]	FNA	Yes	Lymphoma NOS	41 (28/13)	98/100	100	92		SE or CNB
Gong et al. [13]	FNA	Yes	FL grade 1-2 DLBCL	12 9	ND	ND	ND	100 100	SE
McCroskey et al. [14]	FNA	Yes	FL grade 1-2	194	94/ND	ND	ND	99	CNB

*AITL,* angioimmunoblastic T-cell lymphoma; *ALCL* anaplastic large cell lymphoma; *BL,* Burkitt lymphoma; *CFU,* clinical follow-up; *CNB,* core needle biopsy; *DLBCL,* diffuse large B-cell lymphoma; *FL,* follicular lymphoma: *FNA,* fine needle aspiration; *LPL, *lymphoplasmacytic lymphoma; *MCL,* mantle cell lymphoma; *MZL,* marginal zone lymphoma; *ND,* not determined; *NHL, *non-Hodgkin lymphoma; *NOS,* not otherwise specified; *NPV,* negative predictive value; *PL,* plasmablastic lymphoma; *PPV,* positive predictive value; *PTCL,* peripheral T-cell lymphoma; *SE,* surgical excision; *Se* sensitivity; *SLL,* small lymphocytic lymphoma; *Sp,* specificity; *SVB,* small volume biopsy

^$^For MFC; *excluding Hodgkin lymphoma

## What about cytology?

Cytology, including FNAs, has for a long time been held in poor regard among both oncologists and hematopathologists. Back in 2004, Hehn et al. published an article in the *Journal of Clinical Oncology* [15] where they concluded that FNA is “woefully inadequate” for lymphoma diagnosis and may misguide treatment. Since then, and probably even before, there has been sort of a low-intensive war ongoing between the cytology and hematopathologist communities with sparkling battles a little now and then [10, 16–18]. The fact that cytologists are not always full-fledged hematopathologists and vice versa has perhaps contributed to the dispute. Over time, as ancillary techniques (particularly MFC) have matured and proven their role for lymphoma diagnosis on SVBs, the debate is becoming more nuanced. Interestingly, the 2021 guidelines from the American Society for Clinical Pathology and the College of American Pathologists indeed conceded that it cannot be excluded that lymphomas may be reliably diagnosed and subclassified by FNA if relevant ancillary techniques are applied [19]. Ceasefire seems to be within reach with the joint effort of hematopathologists and cytologists to produce the first WHO Reporting System for Lymph Node, Spleen, and Thymus Cytopathology [20].

**Fig. 2:**
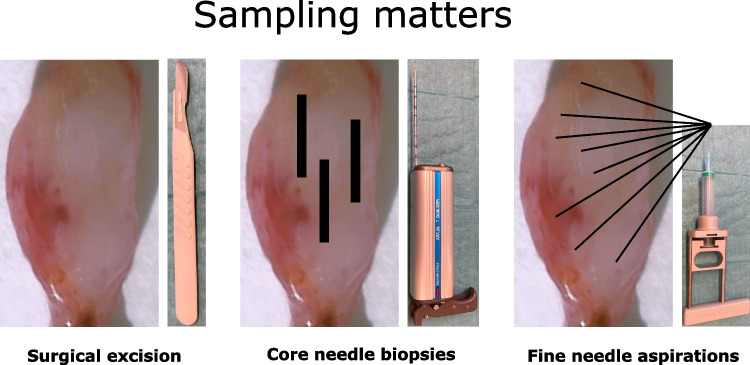
Sampling techniques of lymph nodes. Multiple areas can be sampled with FNA if many needle passes are performed, allowing both immunophenotypical analysis by MFC and cytomorphological evaluation. CNBs allow some architectural assessment. Ideally, FNAs and CNBs can be performed on the same lymph node if SE is not an option

The main disadvantage of FNA cytology is that, in contrast to both CNBs and SEs, the tissue architecture is lost with basically only individual cells to scrutinize. A perhaps underestimated advantage is that multiple areas of the lymph node can be covered if many needle passes can be performed (Fig. 2), which depends on the location and size of the lymph node and the length of the needle. CNBs usually only allow a restricted view of the tissue, as multiple biopsies are less likely to be performed than multiple fine needle passes, which are less traumatic and faster. Therefore, accurately performed FNAs will probably be more representative of a lymph node than CNBs.

## What can flow cytometrists do?

The ideal situation in case of SE is to have the biopsy placed in a gauze compress humidified with saline and rapidly brought to the pathology laboratory. There, depending on the size of the biopsy, a part will be snap-frozen and maintained at -80 °C for further (essentially molecular) studies, a part will be fixed and paraffin embedded, and a part will be dilacerated to make a cell suspension for MFC. During this cutting process, some imprints can also be performed on glass slides for rapid staining and microscopic observation. CNB can be processed the same way, yet with the limitation of size. Instead, concomitant FNA is better, allowing full morphological analysis of the CNB. Several FNAs can be sampled, one being placed in a tube with saline or buffer and processed for MFC [21]. Other types of samples that can be submitted to immunophenotyping are pleural fluid, ascites, vitreous humor, cerebrospinal fluid, and, very informative, the rinse of CNB/FNA needle. The latter is especially useful for the precious brain biopsies [22].

MFC for the diagnosis of lymphomas has developed rapidly after the introduction of this technology [23, 24]. Peripheral blood and bone marrow require a first pre-analytic step of washing with phosphate buffered saline (PBS) to remove plasma immunoglobulins and allow for the determination of monotypy. The latter involves incubation of the cells with antibodies directed against human immunoglobulin light chains kappa and lambda, conjugated to different fluorochromes. The clonality of a B-cell lymphoma will translate to the homogeneous expression of the same isotype by all lymphomatous cells, i.e., monotypy, highly suggestive of clonality. This detection can be applied to other cell suspensions without the washing step. Of note, the cell suspension obtained from disaggregation of a lymph node or other tissue can also be placed in PBS or a richer medium if the sample needs to be saved for a longer time. Monotypy detection, however, has its own caveats. It can be hidden by the presence of two concomitant clones, one kappa and the other lambda. It can result from an ongoing immune response with the transient expansion of a monotypic antigen-specific clone. In such cases, the remaining normal population of B-cells can help. One last deception is that of Castleman disease with the monotypy of polyclonal immunoglobulins as molecular analysis will disclose [25].

Monotypy detection is obviously not enough for proper immunophenotyping of lymphomas and dedicated panels have been developed, with or without light chain detection, allowing to classify lymphoproliferative disorders. These have long been part of the WHO and other recommendations and appear in the most recent classification systems [26, 27]. The major antibodies necessary for a basic panel have been well described [28].

**Fig. 3:**
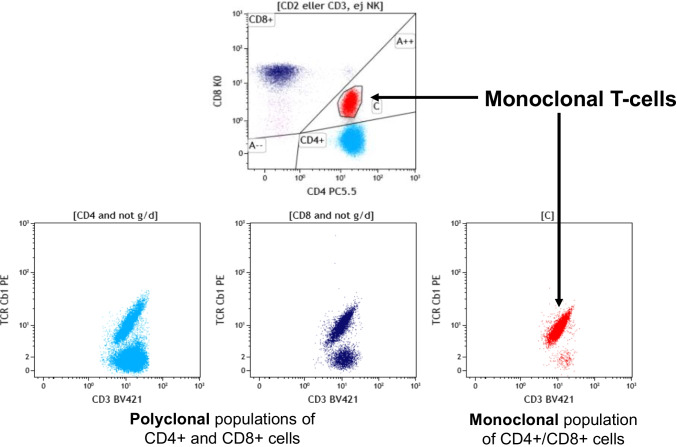
FNA from enlarged lymph node in the neck of a 55-year-old patient. The flow cytometry dot plots reveal monotypic expression of TRBC1. After full diagnostic work-up including surgical excision with histology and immunohistochemistry, this proved to be early involvement of AITL (angioimmunoblastic T-cell lymphoma)

A more recent extremely useful development is that of antibodies to the constant domain 1 of the T-cell receptor (TCR) beta chain (TRBC1). Indeed, two genes code for this domain (TRBC1 and TRBC2), and are randomly selected in a given T-cell during TCR gene rearrangement and therefore identical in all cells of a clone. For many years, PCR for TCR gene rearrangements [29] was the best option to determine clonality of T-cells, thus confirming a T-cell neoplasm, since available corresponding immunophenotypic assays with MFC were quite awkward [30, 31]. With antibodies to TRBC1 and, more recently, TRBC2, determination of T-cell clonality is now easily achievable with MFC [32], and the technique is in diagnostic use at leading hematopathology centers for α-β TCR-positive malignancies [33–36]. This approach allows accurate and rapid diagnosis of most T-cell lymphomas by combining MFC analysis with morphology of SVBs, i.e., FNA and/or CNB (Fig. 3). Perhaps even more importantly, the analysis also reduces the likelihood of a diagnosis of T-cell lymphoma in patients with unclear lymphadenopathy, based on polytypic expression of TRBC1. The agreement between monotypic T-cell populations and T-cell lymphoma is very high whatever the affected tissue reaching 97–100% sensitivity in several studies [33–35]. The reported specificity is also high, from 84% to 100% in recent reports, depending on the tissue examined [34, 37]. Specificity is lower with peripheral blood or bone marrow samples, largely due to the frequent finding of small T-cell clones of unknown significance (T-CUS), which do not equal malignancy [37]. False negatives do occur, albeit rarely, emphasizing that clonality data must be interpreted in the clinical and morphological context. One caveat in using this antibody is that it is necessary to concomitantly test at least for the γ or δ TCR chains (or a combined anti γδ reagent). In case of a clone using a γδTCR, TRBC1 will be negative, which could lead to conclude falsely to a TRBC2 clone. Another way is to also use antibodies that target the αβTCR globally, or specifically recognize TRBC2, the latter having recently become commercially available (Invitrogen, mouse monoclonal SAM.2).

Of course, this ideal situation of combined approaches requires the availability of an MFC platform in short vicinity to the pathology department. Since such platforms routinely perform NHL diagnosis on peripheral blood or bone marrow, they usually have the necessary reagents and can accommodate specific FNA samples assays with minor cost increase.

In summary, MFC presents many advantages. It is fast, results being available within 1 to 3 h. At variance from IHC, it can provide information about numerous markers in a single test. Because of its ability to detect monoclonal populations of both B- and T-cells, it can have a significant impact on CNB diagnoses, especially with limited or inadequate material when architectural or immunohistochemical features are hard to assess. Examples include indolent B-cell lymphomas such as follicular lymphoma (FL) or nodal marginal zone lymphoma and some T-cell lymphomas with follicular T-helper cell phenotype. In such cases, MFC may help to avoid lymph node excision. Current flow cytometers now use 8 to 10 colors, and upcoming instruments are quickly changing the landscape with 12 to 13 colors available. This is largely sufficient to have a reliable answer even from a limited sample. It can discriminate small phenotypically abnormal lymphoid subsets from their normal or reactive microenvironment. Conversely, any notion of tissue architecture will be lost. Samples need to be processed rapidly as fresh material, and for small amounts, it is usually a one-shot method implying that the right panel must be used. Still, MFC is a useful ancillary method.

## Combined cytology and MFC for an integrated approach?

At least for exclusion of a lymphoma diagnosis, FNA combined with MFC is a very attractive approach with a high negative predictive value (Table 1), but the pertinent question is whether FNA also can be used to confidently diagnose lymphomas if relevant ancillary techniques are applied. Some studies have addressed this issue. In one publication examining the diagnostic accuracy of cytology combined with MFC, the overall agreement between cytology/MFC on one hand, and the final histopathological WHO diagnosis from SE on the other, was 87% when 89 consecutive lymphoma cases were examined [11]. Notably, for the most common non-Hodgkin lymphoma subtypes, the concordance rate was 100% for small lymphocytic lymphoma (SLL), 91% for FL and 82% for DLBCL (Table 1). Even higher concordance rates were recorded in an earlier publication (100% for SLL, 100% for FL, and 95% for DLBCL) (Table 1) [10]. In another study, combined MFC and cytologic analysis had a specificity of 100% and a sensitivity of 98% for subtyping B-cell lymphomas when 55 cases with corresponding histology were used as reference standards, translating to a positive predictive value of 100% and negative predictive value of 92% [12]. It should be noted that in this study, both SEs and CNBs appear to have been used as reference histologic material. In addition, there was a selection bias towards patients older than 50 years or with recurrent lymphoma, which may have inflated the high positive predictive value. Looking specifically at FL, the results from some studies are interesting. Grading of FL is based on the number of centroblast-looking lymphoma cells on tissue sections [26, 38] with some modifications in the 5th edition of the WHO classification [27].

**Fig. 4:**
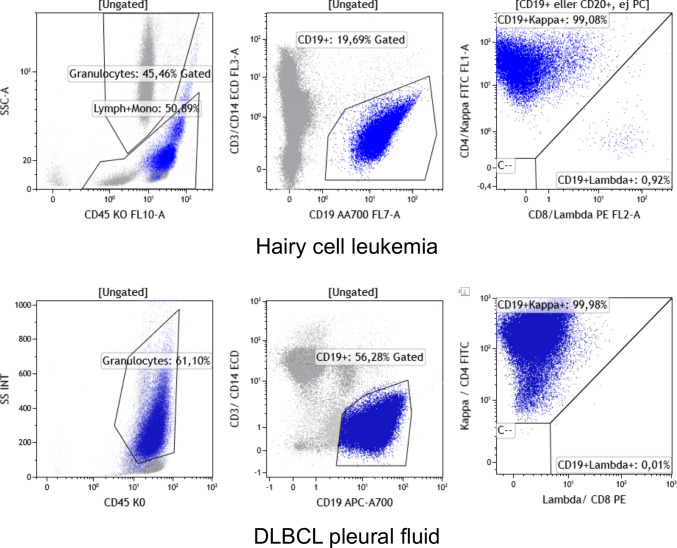
Cell size can be determined by flow cytometry. Examples of large lymphoma cells in flow cytometry as evidenced by side scatter (SSC) on the SSC/CD45 dot plot. B-cells, colored in blue, appear in the monocyte area in the peripheral blood in a case of hairy cell leukemia and in the granulocyte area in pleural effusion of a case of DLBCL

One advantage of MFC, besides its ability to rapidly and accurately phenotype millions of cells, is that side and forward scatter characteristics can be used for determination of cell size. The fact that lymphoma cells sometimes are larger than normal lymphocytes is an important feature to keep in mind when performing MFC. The use of a pan-B (CD19) or pan-T (CD3, CD5 or CD7) marker allows, in a scattergram with this marker in abscissa and side scatter in ordinate, to identify the larger lymphoma cells. For instance, hairy cell leukemia lymphocytes often appear in the monocytic area and cells from DLBCL in the granulocytic area (Fig. 4). Already in 2002, Gong et al. [13] quite convincingly showed that all FL grades 1 and 2 had less than 20% of large cells on MFC, using SEs as gold standard for the final diagnosis. DLBCL and large cell transformation of low-grade B-cell lymphoma could be reliably diagnosed by FNA if more than 40% large cells were present on both cytology and MFC. In general, definitive diagnosis was more difficult with high grade lesions. In a study comprising 342 FLs on CNBs, McCroskey et al. [14] concluded that low grade FLs (defined as grade 1 or 2) can be confidently diagnosed with FNA in combination with MFC with a concordance rate of 99% with the corresponding CNB. Of note, SE was not used as reference standard in this study [14]. Taken together, these studies suggest that a precise lymphoma diagnosis may be possible by FNA and MFC alone (Table 1). Nevertheless, it is important to realize that CNB, FNA, and MFC are complementary methods and most often not substitutable. A limitation of MFC is that some lymphoma cells (especially those from high grade lesions) can be fragile and thus escape detection. Concomitant cytology may reveal corresponding fragile cells with bare nuclei without diagnostic value. In such cases, histological material is necessary. Sometimes, a few viable lymphoma cells from high grade lesions can be detected by MFC despite deceptive cytomorphology with mostly degenerate non-diagnostic cells, thus helping to make a decisive diagnosis. Yet, a significant limitation is that subclassification of many of the reactive lymphadenopathies will be difficult based on cytology and MFC.

## The diagnostic accuracy of different approaches depends on the context

**Fig. 5:**
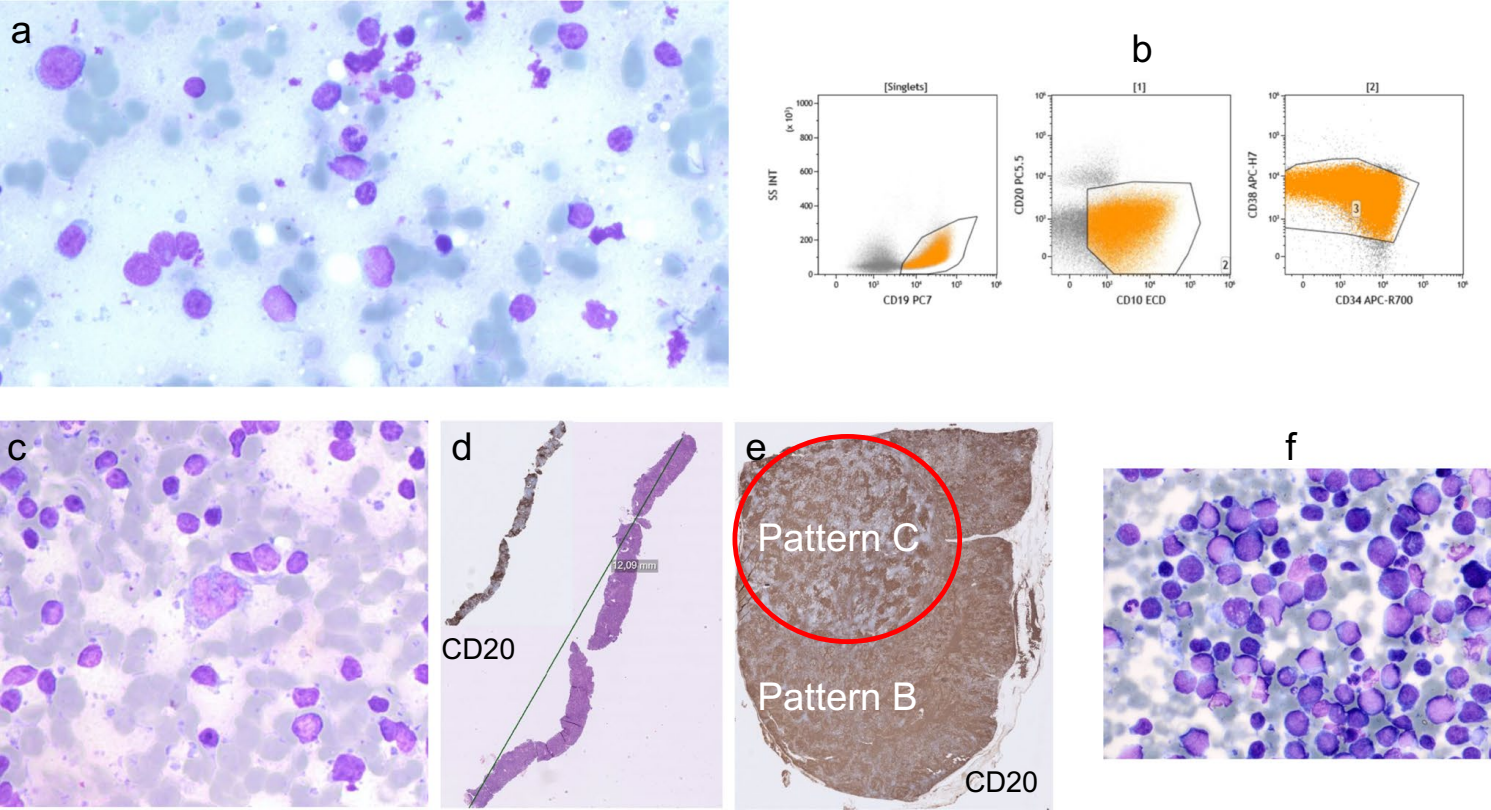
a–b FNA from a parotid lump in a child with precursor B-lymphoblastic leukemia; **a** cytology smear stained with Giemsa; **b** flow cytometry dot plots demonstrating expansion of B-lymphoblasts (orange) with characteristic immunophenotype (CD19+/CD20-/CD10+/CD34+/CD38+); **c–e** Diagnostic specimens from an abdominal tumor in a patient with nodular lymphocyte predominant Hodgkin lymphoma. **c** FNA cytology smear stained with Giemsa; **d** CNB stained with hematoxylin-eosin and for CD20; **e** SE stained for CD20 revealing the prognostic patterns B and C; **f** FNA from a case of diffuse large B-cell lymphoma, non-germinal center type (cytology smear stained with Giemsa). The results from all ancillary techniques (MFC and cell block with IHC, in situ hybridization for EBV, and FISH for *MYC* gene rearrangements) revealed CD20+/CD19+/kappa+/bcl2+/CD10-/bcl6+/mum1+/CD5-/cyclinD1-/EBV-/cmyc- lymphoma cells without *MYC* gene rearrangement (not shown)

Diagnostic hematopathology relies on four main pillars, namely morphology, immunophenotyping, molecular genetics, and the clinical setting. Yet, the relative importance of these diagnostic tools in rendering a specific diagnosis is not static, may change over time as diagnostic techniques evolve, and ultimately depends on the specific diagnosis itself. As the ancillary techniques are getting more and more powerful to obtain critical diagnostic information, it comes naturally that in some situations and for certain types of diagnoses, less emphasis will be put on morphology. A striking example from hematology is the lack of importance of ring sideroblasts in the context of myelodysplastic syndromes with *SF3B1* mutation, which today defines a specific entity in the 2022 ICC and updated WHO classifications [26, 27]. The term ring sideroblasts is fading away from the classifications along with the challenge to find them (notably if mutational analysis is available up-front). This change of perspective may also come true for certain lymphoma subtypes as molecular genetic information allows relevant diagnostic subclassification [39, 40]. Already, there are some lymphoma entities where cytomorphology or histology adds little in the face of flow cytometric immunophenotyping and genetic analysis. For example, consider the FNA cytology from a 2-cm large lump in the parotid region of a 4-year-old child presenting with fatigue (Fig. 5a). The cytomorphology was difficult to interpret and labeled as atypical. However, MFC immediately revealed a prominent population of CD19-, CD10-, and partly CD34-positive B-lymphoblasts (Fig. 5b), and a final diagnosis of precursor B-lymphoblastic leukemia could be established on the subsequent bone marrow investigation.

By contrast, there are entities that will probably always require histology, at least in the foreseeable future. An example is shown in Fig. 5c–e with some details from the diagnostic work-up of a 34-year-old patient presenting with ankle joint and wrist arthritis, erythema nodosum, hypercalcemia, and a tumor in the abdomen. In this case, a FNA from the abdominal tumor did not reveal any monoclonal B-cells or aberrant T-cells by MFC. The large cell among the small lymphocytes on cytology (Fig. 5c) would perhaps not escape detection by the experienced cytopathologist, but a frank diagnosis of nodular lymphocyte predominant Hodgkin lymphoma (NLPHL) would be very challenging, to say the least, even with the use of cell blocks to determine the immunophenotype. This is because the overall architecture is necessary for diagnosis and exclusion of other entities such as TCHRLBCL. The neoplastic cells in TCHRLBCL can look very much like Hodgkin cells in NLPHL (Fig. 5c), and both types most often escape detection by MFC because of their low number. The differential diagnosis can also be quite challenging on a CNB. In addition, neither cytology nor CNBs (Fig. 5d) would be likely to decipher the different histologic NLPHL patterns of prognostic value [26, 27] (Fig. 5e). Hence, to diagnose NLPHL and TCHRLBCL (Fig. 1), histologic material is necessary, and surgical biopsies are preferable. For other diagnoses, it may be debatable whether histologic material (SE or CNB) is necessary for full subclassification. The DLBCL shown in Fig. 5f (overwhelming number of large blast-like cells on cytology) leaves little doubt as to the final diagnosis when the full work-up had been performed including MFC and in situ hybridization for EBV RNA and FISH for *MYC* gene rearrangements on a cellblock, thus revealing CD20+/CD19+/kappa+/bcl2+/CD10-/bcl6+/mum1+/CD5-/cyclinD1-/EBV-/cmyc- lymphoma cells without *MYC* gene rearrangement (not shown). Nevertheless, changing the prerequisites to germinal center phenotype (CD10+ instead of CD10-/bcl6-/mum1+) would make it difficult to rule out FL grade 3B. Admittedly, in many cases of DLBCL, when the fraction of large cells falls within the grey zone (> 20% by MFC and < 40% by either MFC or cytomorphology) where both low-grade and high-grade lymphomas are likely to be found,^37^ full diagnosis on cytology/MFC is simply not trustworthy. Clearly then, whether a full subtype diagnosis of lymphoma is possible on cytology/MFC not only depends on the specific diagnostic criteria on histology, but also on available ancillary techniques. Table 2 shows a debatable list of most lymphoma subtypes for which cytology with appropriate ancillary techniques may be sufficient or not for diagnosis, based on data from the literature [10, 11, 13, 14, 26, 27, 41–44]. Although the approach with FNA and MFC alone is uncontroversial for certain B-lymphoproliferative disorders including primary effusion lymphoma, lymphoblastic lymphoma, and hairy cell leukemia [26, 27], it cannot be recommended for lymphoma subtypes relying on tissue architecture for diagnosis and/or lymphomas with a large inflammatory component. Hence, subtyping Hodgkin lymphomas or T-cell lymphomas is best avoided with FNA and MFC alone (Table 2). On the other hand, it may be reasonable to diagnose other lymphomas with less dependence on architectural features that instead rely more on characteristic cytomorphology, specific immunophenotypic profiles, and objective measurements of cell size with MFC. Such lymphomas include Burkitt lymphoma, anaplastic large cell lymphoma, mantle cell lymphoma, small lymphocytic lymphoma, FL grades 1–2, and some cases of DLBCL (Table 2). Lymphoplasmacytic lymphoma and nodal marginal zone lymphoma are controversial because of their less characteristic immunophenotype, but other ancillary techniques may help, e.g., paraprotein and *MYD88* mutation analysis (Table 2). Finally, it should be noted that a limited focus of large cell transformation of indolent B-cell lymphomas will be missed without histology (Table 2).

**Table 2:**
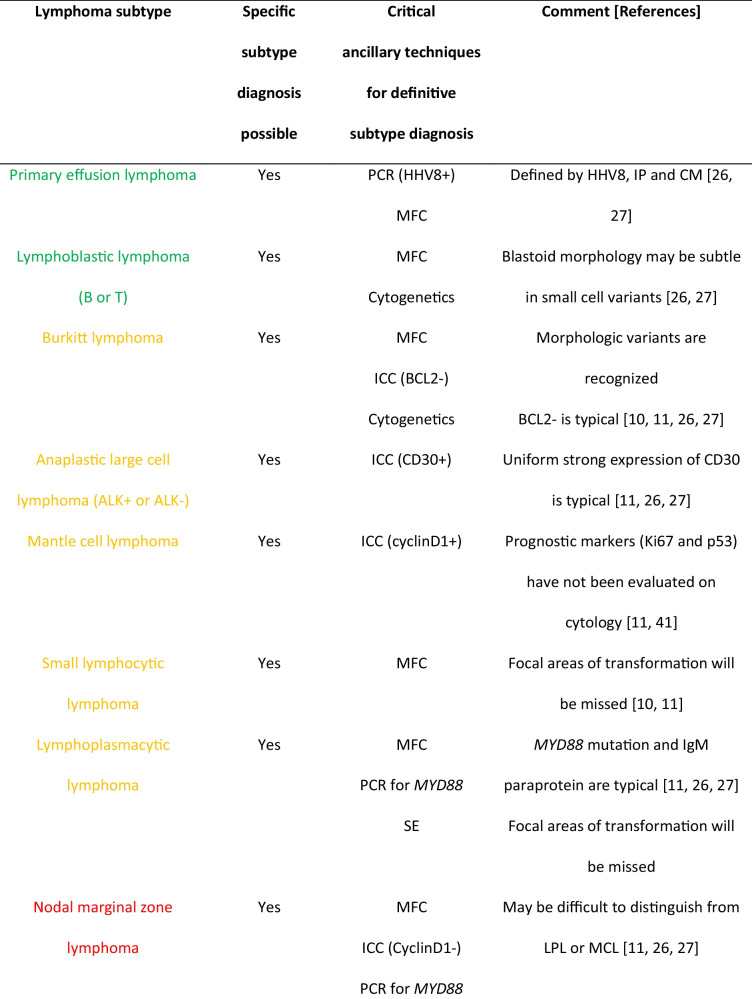

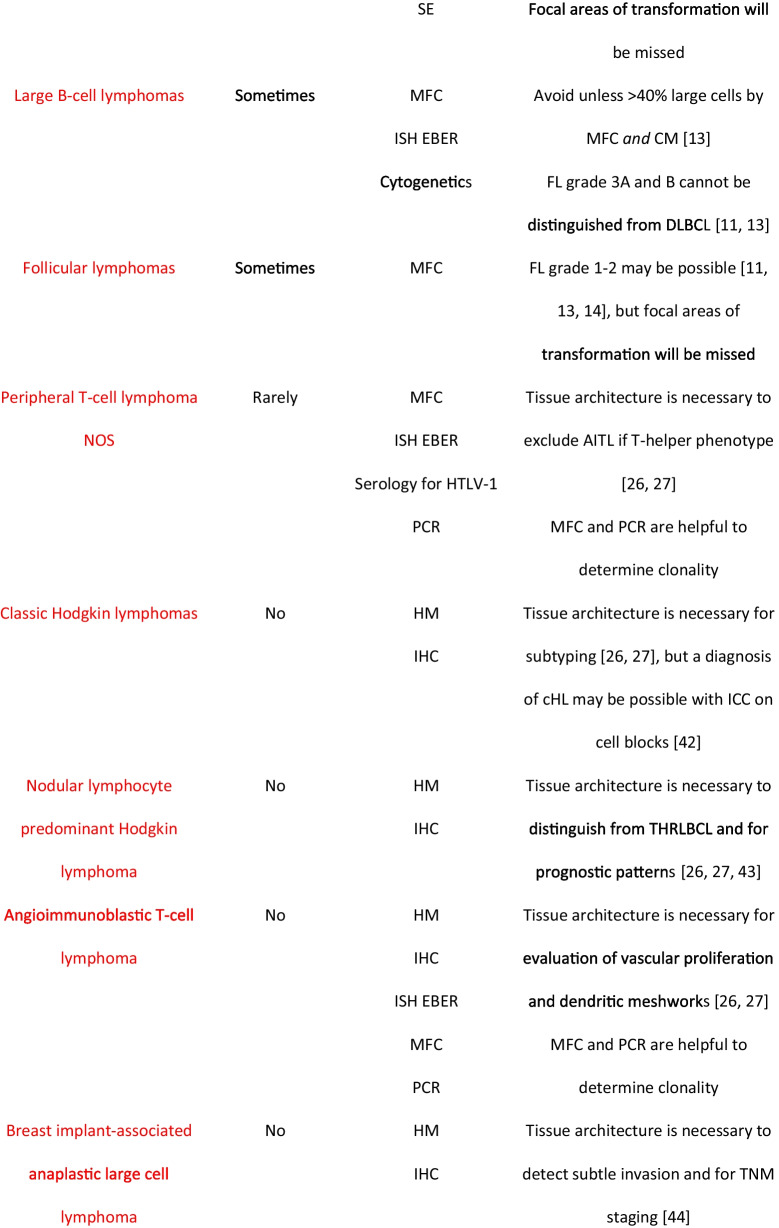
Debatable list of lymphomas for which cytology with appropriate ancillary techniques may be sufficient or not for subtype diagnosis in increasing order of controversy.  ;  ;

AITL, angioimmunoblastic T-cell lymphoma; cHL, classic Hodgkin lymphoma; CM, cytomorphology; DLBCL, diffuse large B-cell lymphoma; FL, follicular lymphoma; HHV8, human herpesvirus 8; HM, histomorphology; ICC, immunocytochemistry on cell block; IHC, immunohistochemistry; IP, immunophenotype; ISH EBER, *in situ* hybridization for Epstein-Barr virus; LPL, lymphoplasmacytic lymphoma; MCL, mantle cell lymphoma; MFC, multiparameter flow cytometry; PCR, polymerase chain reaction; SE, serum electrophoresis; THRLBCL, T-cell/histiocyte rich large B-cell lymphoma; TNM, tumor node metastasis

## Future perspectives

Given the strength of MFC to exclude or confirm B- or T-cell lymphomas, it should be included in the routine work up of lymphadenopathies. A surgical biopsy should be the first option whenever possible. If this is not feasible, multiple CNBs for histology with concomitant FNAs for MFC can be performed, usually by ultrasound or sometimes CT guidance. The organization for the diagnostic work-up varies considerably depending on local or national traditions. For example, in France and Germany, MFC facilities often reside outside pathology departments resulting in less MFC being performed on SVBs. In Sweden, MFC is often incorporated within (hemato)pathology facilities and routinely performed to exclude or confirm lymphoma. In the USA, hematopathologists routinely perform MFC in tissue although not all perform MFC in CNBs. At some institutions, CNBs with concomitant FNAs for MFC is the established practice, as it is in Sweden (Fig. 2) [14].

Machine learning has so far begun to be applied to Artificial Intelligence (AI)–aided diagnosis through imaging techniques, of note using SE samples [45] or tissue arrays prepared from SE [46]. Interestingly, MFC on FNAs was used in a model assessing ex vivo drug sensitivity in 261 samples from canine lymphoma [47]. A convoluted neural network classifier was also developed from MFC data of suspected Hodgkin lymphoma by Simonson et al. [48] on more than 1000 human CNB and FNA samples. These still limited and recent examples, with already good and certainly improvable results, suggest that AI will probably find its way in this specific field of MFC. Analysis of the clonal hierarchy and of the microenvironment within lymph nodes could be interesting to assess with AI clustering software [49]. Indeed, lessons could be learned for instance from the dissection of the normal erythroid lineage with the robust and rapid tool FlowSOM [50].

Although the current lymphoma classifications [26, 27] do not take gene mutations into account (with a few exceptions), they are very likely to impact future classification schemes. For example, the most common lymphoma type, i.e., DLBCL, can be subdivided into molecular subgroups reflecting the pathogenesis. Several investigators have independently and with different approaches found distinct molecular subtypes based on point mutations and copy number alterations with remarkable overlap, suggesting a solid biological basis for molecular subclassification [39, 40, 51]. In this context, cytological specimens, including archived slides, can be a valuable source for comprehensive molecular testing, provided that enough cells are collected [52–54]. In fact, cytological specimens prepared by various methods are often equivalent or even superior to small biopsies and cell blocks [55, 56]; one advantage compared to paraffin-embedded tissue is the high DNA quality lacking formalin-fixation induced artefactual mutations when sequencing [57]. With careful FNA, 100 ng DNA can easily be obtained which is usually enough for molecular studies such as next generation sequencing (NGS). Along with the ongoing characterization of the mutational landscape of lymphomas revealing subtype-specific patterns [58–60], algorithms using molecular signatures are likely to boost subclassification on SVBs. One may argue that SE is always better, not least for research purposes, but this vision will inevitably collide with reality, as forces outside the pathology community tend to favor rapid and cheap diagnostic approaches. Although far from devoid of subjectivity, ancillary techniques are probably more objective than cytomorphology, which in the future may shift the balance among the diagnostic tools in favor of NGS and MFC.

## Conclusions

MFC should be an integrated part of the diagnostic work-up of lymphadenopathies, particularly when core needle biopsies and/or fine needle aspirations constitute the diagnostic material. In the current era of precision medicine, when hematopathologists paradoxically are facing less material for diagnostic and research purposes, alternative strategies need to be developed to perform critical immunophenotypic and molecular analyses. FNA combined with ancillary techniques including MFC and molecular genetic analyses represent one possible and probably underestimated avenue. A specific subtype diagnosis of lymphoma is possible for many cases with cytology combined with MFC, and more diagnoses on cytology will probably be made with the development of flow cytometric tools such as TRBC1 restriction analysis and molecular techniques such as NGS for mutation detection. We are likely to become more dependent on MFC to compensate for small volume material, and we are just on the threshold of routine NGS analyses on lymphoma samples.
